# Hybrid Multi-Objective Neural Architecture Search for Lightweight Patch-Based Mistletoe Classification in UAV Imagery

**DOI:** 10.3390/jimaging12070281

**Published:** 2026-06-26

**Authors:** Miguel-Angel Gil-Rios, Nivia Escalante-Garcia, Juan C. Valdiviezo-Navarro, Paola Andrea Mejia-Zuluaga, León Dozal, Ivan Cruz-Aceves

**Affiliations:** 1 Departamento de Tecnologías Emergentes Industriales e Informáticas, Universidad Tecnológica de León, León 37670, Guanajuato, Mexico; mgil@utleon.edu.mx; 2 Laboratorio de Iluminación Artificial, Tecnológico Nacional de México/IT Pabellón de Arteaga, Pabellón de Arteaga 20670, Aguascalientes, Mexico; nivia.eg@pabellon.tecnm.mx; 3 EPM SECIHTI–Centro de Investigaciones en Óptica, A.C., Unidad Aguascalientes, Aguascalientes 20200, Aguascalientes, Mexico; 4 SECIHTI-CentroGeo, Mérida 97302, Yucatán, Mexico; 5 Centro de Investigación en Ciencias de Información Geoespacial (CentroGeo), Ciudad de México 14240, CdMx, Mexico; pmejia@centrogeo.edu.mx; 6 SECIHTI-CentroGeo, Aguascalientes 20313, Aguascalientes, Mexico; leon.dozal@gmail.com; 7 SECIHTI-Centro de Investigación en Matemáticas, A.C., Guanajuato 36000, Guanajuato, Mexico; ivan.cruz@cimat.mx

**Keywords:** network architecture search, lightweight, multi-objective evolutionary algorithm, decomposition, mistletoe, classification

## Abstract

This paper proposes a novel method for automatically designing lightweight Convolutional Neural Network (CNN) architectures. (1) Background: Automated remote sensing for vegetation monitoring faces challenges from structural complexity and cluttered backgrounds. For detecting parasitic *Phoradendron velutinum* infestations, existing vision frameworks rely on handcrafted, overparameterized CNNs, limiting deployment on localized edge computing platforms. (2) Methods: To address this efficiency-accuracy trade-off, a two-phase hybrid multi-objective Neural Architecture Search (NAS) strategy is implemented. First, the Multi-Objective Evolutionary Algorithm based on Decomposition (MOEA/D) minimizes classification error and the number of trainable parameters. Second, an Iterated Local Search (ILS) metaheuristic refines promising non-dominated solutions. The approach was evaluated using cost-effective aerial RGB imagery, processing a balanced dataset of 5000 patches (64×64 pixels) under a rigorous three-way data partition to prevent data leakage. (3) Results: The discovered 10-layer CNN topology achieved high feature-extraction efficiency. On the unseen testing set, the model yielded an Accuracy and F1-Score of 0.979, a Precision of 0.982, a Recall of 0.976, and a Jaccard Index of 0.958, outperforming the compared models. Operating with only 2040 trainable parameters, the optimized architecture establishes a highly viable paradigm for real-time digital image processing on hardware-constrained monitoring devices.

## 1. Introduction

**Figure 1:**
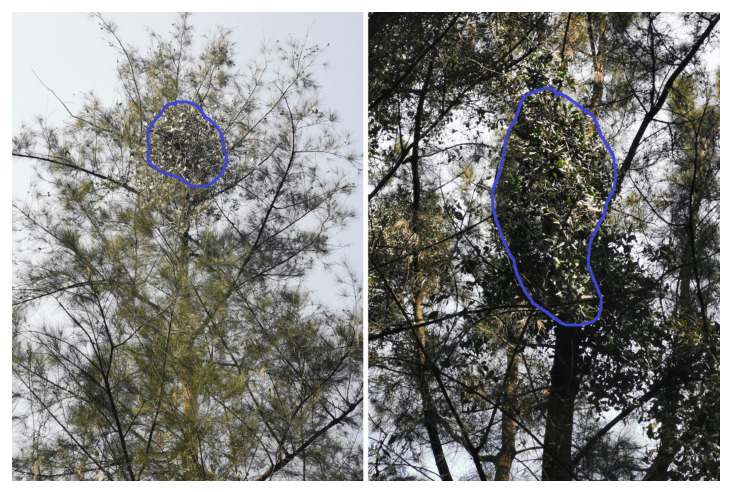
Two cases of host trees, located in an urban park of México City, dominated by a mistletoe species known as *Struthanthus Interruptus*. The blue line in both figures highlights the mistletoe infestations.

Forest decline is increasingly driven by interactions between climatic stressors and biotic agents, leading to severe impacts on stand structure, biodiversity, carbon dynamics, and ecosystem services [1]. Among these biotic agents, mistletoe infestation has become a persistent driver of canopy deterioration in both forested and semi-managed ecosystems [2,3,4]. As hemiparasitic plants, mistletoe species possess chlorophyll and perform photosynthesis, but lack a conventional root system to absorb water and nutrients directly from the soil. Hence, they develop specialized structures called haustoria that penetrate host branches to intercept xylem sap. In the early stages of infestation, the parasite typically blends into the host foliage, making visual detection challenging. Conversely, in advanced stages, the mistletoe dominates the tree canopy, severely reducing host vitality and accelerating dieback. Figure 1 illustrates two urban trees infested with the mistletoe species *Struthanthus interruptus*.

Mistletoe infestations cause host vigor, crown degradation, growth suppression, and, under prolonged stress, increased host-tree mortality, particularly in regions already experiencing drought and climate variability [5,6]. For example, a forest inventory in México City identified mistletoe as a primary driver in tree decline, with infestations affecting more than 48% of urban trees [7]. In these contexts, prompt identification of highly compromised or dead trees is essential for understanding decline dynamics and for informing silvicultural decisions, conservation planning, and operational forest monitoring [6].

Conventional field surveys are limited in their capacity to assess mistletoe-related decline due to many symptoms manifest in the upper canopy, making them difficult to observe from the ground. Consequently, distinguishing between mild infestation, advanced decline, and tree mortality remains a critical challenge during ground-based inspections. High-resolution imagery acquired from Unmanned Aerial Vehicles (UAVs) offers a robust alternative for multi-scale remote sensing observations. Forest monitoring in this context spans a continuum, ranging from landscape-level mapping to individual tree crown (ITC) assessments. At the finest scale of this continuum, patch-based classification serves as the foundational analytical unit. By extracting and classifying localized sub-images, UAV-based frameworks can effectively isolate discoloration, dieback, and other canopy symptoms within ITCs that are not detected on the ground [8,9,10].

Despite this potential, translating UAV imagery into adaptable diagnostic tools remains challenging. Mistletoe-related symptoms manifest as subtle, heterogeneous, and spatially variable canopy patterns, necessitating models capable of distinguishing fine-grained features within highly complex backgrounds. Although deep learning models have demonstrated strong performance in forest health monitoring workflows, this trade-off is critical; real-world deployment depends heavily on both classification performance and computational efficiency, which are governed by parameters such as a memory footprint, inference time, and floating-point operations (FLOPs) [11,12,13].

To balance discriminative performance with low computational overhead, lightweight deep learning architectures have gained considerable attention [11,12,13]. However, manually engineering these topologies for ITC classification is an arduous task, particularly when balancing predictive power against strict hardware constraints. Neural Architecture Search (NAS) mitigates these limitations by automating the discovery of candidate networks personalized to explicit design criteria [14,15]. In deployment-oriented applications, NAS is particularly advantageous when formulated as a multi-objective optimization problem, allowing the simultaneous evaluation of classification accuracy and resource efficiency [16].

Recent research has advanced forest health monitoring through the integration of UAV-based imagery and artificial intelligence, employing techniques such as deep learning [17,18] and genetic programming [9,19]. For instance, these frameworks have successfully mapped diverse forest threats, including bark beetle infestations [20], while convolutional neural networks (CNNs) have been deployed for automated individual tree classification [21]. Furthermore, Mejia et al. [9] applied genetic programming to detect mistletoe infestations in conservation forests, demonstrating the feasibility of automated spectral index discovery. Despite these advances, the integration of deep learning and multi-objective NAS specifically optimized for patch-based mistletoe classification remains unexplored within the remote sensing continuum. This limitation introduces critical bottlenecks in operational workflows, where patch-level inference serves as the foundational computational mechanism for upscaling to crown-level and landscape-scale assessments under stringent hardware constraints.

In order to address the previous challenges, this paper introduces a hybrid multi-objective NAS framework specifically designed for lightweight, patch-based mistletoe classification using high-resolution UAV imagery. The methodology operates on the hypothesis that a hybrid metaheuristic approach, integrating decomposition-based evolutionary optimization with localized iterative search, can systematically discover compact convolutional topologies that minimize both patch-level classification error and parameter complexity. Ultimately, this framework delivers operationally viable models for resource-constrained edge devices while preserving high discriminative accuracy.

Therefore, the main contribution of this work is a hybrid multi-objective methodology that automatically discovers lightweight CNN topologies optimized for patch-based mistletoe classification. The proposed framework successfully identifies highly compact networks that minimize both classification error and parameter complexity. The resulting converged architecture achieves competitive generalization capabilities on unseen testing data while maintaining an exceptionally low computational footprint and reduce inference latency. Consequently, this approach establishes an operationally viable paradigm for integration into geographical information systems and real-time UAV image processing routines.

The remainder of the paper is organized as follows. Section 2 establishes the foundational concepts of CNN architectures and NAS, including the underlying evolutionary metaheuristics and evaluations metrics. Section 3 provides a comprehensive breakdown of the proposed hybrid multi-objective evolutionary method, alongside a detailed description of the UAV image database. The experimental setup, empirical results, and a rigorous discussion on mistletoe classification are presented in Section 4. Finally, Section 5, concludes the manuscript by summarizing the core findings, identifying current limitations, and outlining directions for future work.

## 2. Background

### 2.1. Convolutional Neural Network

Convolutional Neural Networks (CNNs) automate hierarchical feature extraction through a sequence of functional layers [22,23]. A standard CNN topology sequentially integrates functional layers to map input tensors into discriminative feature spaces foundational feature extraction occurs within the convolutional layers through learnable kernels. Formally, for a discrete 2-D image *I* and a localized kernel *K* centered at (0,0), the cross-correlation operation, conventionally termed as convolution in deep learning frameworks, is defined as:

(1) $$G\left(x,y\right)=\sum_{m=-a}^{a}\sum_{n=-b}^{b}I\left(x+m,y+n\right)K\left(m,n\right),$$

where G(x,y) represents the resulting feature map response. To introduce non-linearity, activation functions are systematically applied. The standard Rectified Linear Unit (f(x)=max(0,x)) maps negative inputs to zero, introducing a localized sparsity that mitigates the vanishing gradient problem. Alternatively, Leaky ReLU (f(x)=max(αx,x), with a small constant α) prevents the “dead neurons” phenomenon by maintaining a small, non-zero gradient for negative inputs.

**Figure 2:**
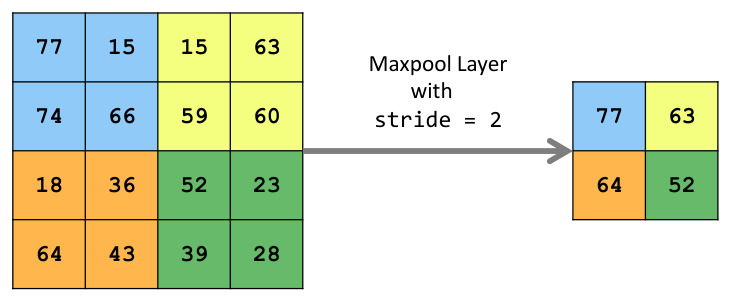
Representation of the maxpool operation using a stride value of 2.

Other architectural components include normalization layers to accelerate convergence [24] and max-pooling operations for spatial dimensionality reduction (see Figure 2). Furthermore, dropout layers are incorporated as a stochastic regularization mechanism during training, randomly deactivating a fraction of neurons to prevent feature co-adaptation and overfitting. Finally, flattening layers map these regularized spatial features into continuous vectors for final classification.

### 2.2. Neural Architecture Search (NAS)

Constructing application-specific CNN models is a non-trivial task due to the vast combinatorial space of configurations. Consequently, NAS has emerged as an alternative to hand-crafted models [25,26]. In NAS, a CNN topology is modeled as a directed acyclic graph (DAG) where nodes represent data states and edges correspond to discrete operations [27]. The state progression of a *t*-th node is defined as:

(2) $$s^{\left(t\right)}=O^{\left(t\right)}\left(s^{\left(t-1\right)}\right),$$

where $O^{\left(t\right)}$ belongs to a predefined set of operators O (e.g., convolutions, pooling, skip-connections). The optimization process evaluates these candidates by balancing classification effectiveness against structural complexity, often quantified by the number of trainable parameters.

### 2.3. Multi-Objective Evolutionary Algorithm Based on Decomposition

The multi-objective evolutionary algorithm based on decomposition (MOEA/D) solves multi-objective problems (MOPs) by decomposing them into *N* scalar subproblems optimized in parallel [28]. Instead of traditional Pareto-dominance selection, new candidate solutions are evolved through crossover and mutation within the proximity of weight vectors $\Lambda =\{\lambda_{1},\ldots ,\lambda_{N}\}$.

To evaluate offspring, objective vectors are transformed into scalar values. Under the Tchebycheff approach, the scalar fitness of a solution *x* for the *j*-th subproblem is defined as:

(3) $$g^{tch}\left(x_{j}|\lambda_{j},z^{*}\right)=\max_{k=1}^{m}\{\lambda_{jk}|f_{k}\left(x_{j}\right)-z_{k}^{*}\left|\right\},$$

where *m* is the number of objectives, $\lambda_{jk}$ is the weight assigned to the *k*-th objective, $f_{k}\left(x_{j}\right)$ is the objective value, and $z^{*}=\left(z_{1}^{*},\ldots ,z_{m}^{*}\right)$ represents the reference ideal point ($z_{k}^{*}=\min_{i=1}^{N}f_{k}\left(x_{i}\right)$ for minimization). The MOEA/D workflow is described in Algorithm 1.
**Algorithm 1:** MOEA/D Pseudocode
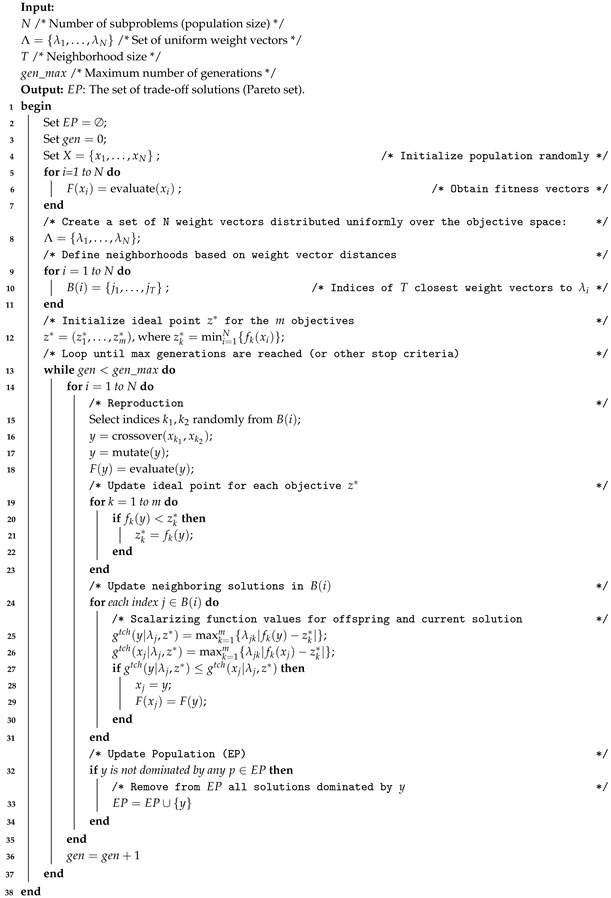


### 2.4. Iterated Local Search

The Iterated Local Search (ILS) metaheuristic circumvents the local optima limitations of traditional local search methods [29,30]. As detailed in Algorithm 2, ILS alternates between an intensive local search refinement phase and, a randomized perturbation mechanism, exploring broader space regions until a convergence criterion is satisfied.
**Algorithm 2:** Iterated Local Search pseudocode
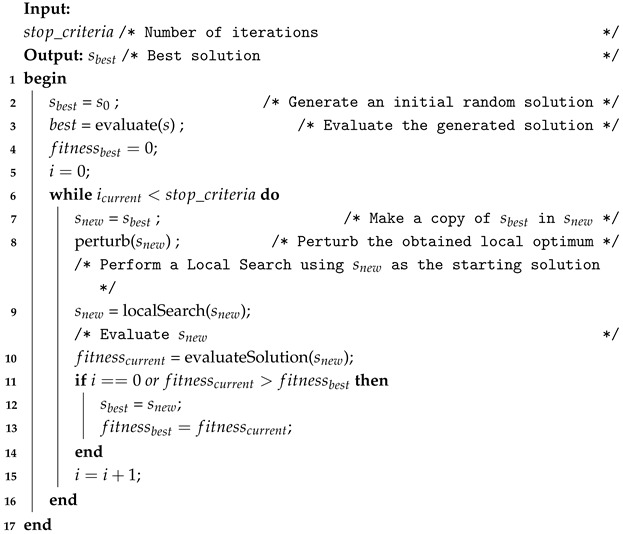


### 2.5. Performance Metrics

To evaluate mistletoe classification performance and structural complexity, models are assessed based on the total number of trainable parameters and five metrics derived from True Positives (TP), True Negatives (TN), False Positives (FP), and False Negatives (FN): Accuracy, Precision, Recall, Jaccard Similarity Coefficient (JSC), and F1-Score. These metrics are computed as follows:

(4) $$Accuracy=\frac{TP+TN}{TP+TN+FP+FN}$$

(5) $$Precision=\frac{TP}{TP+FP}$$

(6) $$Recall=\frac{TP}{TP+FN}$$

(7) $$JSC=\frac{TP}{TP+FP+FN}$$

(8) $$F1=\frac{2\times Precision\times Recall}{Precision+Recall}$$

## 3. Proposed Method

This section presents the proposed hybrid multi-objective metaheuristic. It introduces the CNN architecture encoding strategy, outlines structural constraints to prevent inconsistent models, and describes the methodology for identifying optimal CNN architectures for mistletoe infestation classification.

### 3.1. CNN Model Encoding

Encoding CNN architectures for metaheuristics is crucial for advancing NAS, highlighting its importance within the community. Each solution, or genotype, explores a high-dimensional search space consisting of various layer types, hyperparameters, and tuning requirements while avoiding structural inconsistencies. The design constraints applied ensure that candidate solutions are both valid and effective.

- Linear Feed-Forward approach. Since CNN architectures are primarily encoded as binary vectors, models following a linear feed-forward structure are straightforward to represent as candidate solutions. This vector-based representation set up direct manipulation in accordance with metaheuristic search principles [31]. Consequently, identifying and resolving inconsistent architectural designs is simpler than in non-linear CNN counterparts.
- Minimum Layer Requirements. Every model must include at least one convolutional layer, as an architecture without convolution layers is impractical for this application.
- Kernel Size in convolution layers. Kernel sizes are restricted to 3×3 and 5×5, as these smaller filters are more effective than larger ones [32]. They allow for deeper architectures with fewer parameters while maintaining an adequate receptive field.
- Filter Scaling. The number of filters is specified as a multiple of $2^{n}$, where *n* is constrained to have a discrete value in range [1,5]. Under this constraint, the number of filters is limited to 2, 4, 8, 16, and 32, which optimizes processing efficiency during GPU-accelerated training [33].
- Forward Information Reduction. CNN models achieve higher classification performance by progressively discriminating information through subsequent layers. In the proposed method, this reduction is achieved by employing specific stride values in the convolutional layers and incorporating max-pooling layers [34,35].
- Classification Layers. Flattening, Dense, and SoftMax layers are integrated at the end of the architecture to transition from feature extraction to final classification.

**Figure 3:**
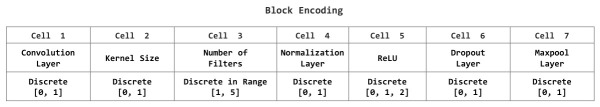
Architectural encoding block illustrating the set of available layers that can be selectively activated to construct a new CNN model.

Using CNN distinct layer types such as convolution, normalization, ReLU/Leaky ReLU, dropout and by applying the previously described constraints, a single processing block can be formed as illustrated in the Figure 3.

Figure 3 outlines a processing block for configuring CNN layers. The first cell indicates whether to add a convolutional layer (1 for yes, 0 for no), while the second cell sets the kernel size to 3×3 for 0 and 5×5 for 1. If the first cell is 1, the third cell defines the number of filters as $2^{n}$, with *n* ranging from 1 to 5. The fourth cell adds a normalization layer if its value is 1, and the fifth cell specifies the type of activation layer: none for 0, Leaky ReLU for 1, and ReLU for 2. The sixth cell controls the addition of a dropout layer with a 0.5 rate if it is 1, and the final cell manages the max-pooling layer.

**Figure 4:**
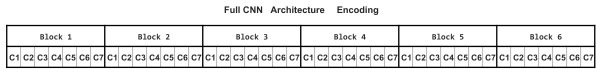
Full CNN encoding involving 6 processing blocks. Consequently, each block is formed by 7 cells representing distinct CNN layer types and parameters setup.

The max-pooling layer reduces the size of the data for the next stages. For example, an initial input of 64×64 is downsampled to 32×32 in the first block. The second through sixth blocks then continue to reduce the size to 16×16, 8×8, 4×4, 2×2, and finally to 1×1. Figure 4 shows the full architecture, which includes the encoding blocks and cells, along with the methods used to create potential solutions.

Following this encoding strategy, each individual is represented as a 42-element vector, assuring compatibility with metaheuristics such as MOEA/D and ILS.

### 3.2. Hybrid Multi-Objective Method for the Neural Architecture Search Process

The architectural configurations of a single block are defined by its internal variables: five cells each have binary states (yielding $2^{5}=32$ combinations), one cell can take on five discrete states, and another cell can assume three discrete states. As a result, a single block can generate 32×5×3=480 unique configurations. Since the network topology replicates this structure across six sequential blocks, the overall size of the search space is $480^{6}$, which equals approximately $1.223\times 10^{16}$ or about $2^{54}$ candidate solutions.

Given the high dimensionality of the search space, metaheuristics are crucial. The proposed hybrid multi-objective method consists of two stages. First, MOEA/D explores the search space to generate a set of non-dominated solutions. Due to the computational demands of training CNN models and restrictions on population size, the number of training epochs is kept minimal to ensure timely competitive results.

**Figure 5:**
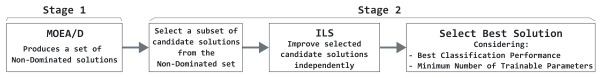
Flow schema of the proposed method consisting of two major stages.

In the second stage, a subset of solutions from the center of the Pareto front generated by the MOEA/D is selected for refinement. This selection targets the knee region, balancing minimizing classification error with parameter complexity. The candidate topologies are then enhanced through a rigorous local optimization process using the ILS metaheuristic, improving their performance beyond the initial search. Figure 5 illustrates the flow schema for this hybrid multi-objective method.

The proposed method combines population-based exploration with localized refinement, requiring specific configurations for both metaheuristics. The MOEA/D stage involves setting the population size and number of generations, while the ILS stage focuses on defining local search iterations and selecting candidate architectures for refinement. Additionally, evaluating each deep neural network solution requires specifying training hyperparameters like epochs, validation frequency, and batch size.

To unify these two optimization paradigms, Iterated Local Search (ILS) uses a strict Pareto dominance criterion instead of traditional multi-objective methods like weighting or weak dominance schemes. While scalarization requires arbitrary assignments, which can introduce bias and limit navigation in non-convex areas, weak dominance may allow improvements in one objective at the cost of another.

The strict dominance scheme adopted here stipulates that a new neighborhood solution is accepted during local search only if it improves at least one objective without worsening any other objective. This approach offers a significant operational advantage for deep network refinement: it eliminates the need for parameter tuning at the hybridization interface, allowing ILS to function as a conservative optimization engine. As a result, this strategy ensures that the localized search effectively drives the selected knee-front topologies toward better convergence. This minimizes the complexity of CNN architectures while maintaining high classification performance.

### 3.3. Materials

The image database for this research was created through a collaboration between CentroGeo and the México City Environment Secretariat (SEDEMA-CORENA) to detect mistletoe in urban parks using remote sensing and artificial intelligence. High-resolution aerial imagery was captured over the San Bartolo Ameyalco ecological conservation forest, located at $19^{\circ }20^{\prime }$ N and $99^{\circ }16^{\prime }04^{\prime \prime }$ W. This area features a variety of coniferous species, mainly *Abies religiosa* and associations of *Abies*, *Pinus*, and *Quercus* [36], which are impacted by the hemiparasitic mistletoe *Phoradendron velutinum*. The mistletoe has a yellowish, velvety appearance in its early stages and penetrates host branches using a haustorium to extract water and nutrients [9,37]. Initially, its foliage blends with the host canopy, but as it matures, particularly during the flowering phase, it becomes structurally and spectrally distinct due to yellowish-green pigmentation from flavonoids, chlorophylls, and carotenoids, with oval-shaped leaves forming dense clusters up to 80 cm long.

Aerial imagery was captured on 13 April 2021, at 12:30 PM (solar elevation: $57.58^{\circ }$, azimuth: $103.45^{\circ }$) using a DJI P4 Multispectral UAV (DJI Co., Ltd., Shenzhen, China). This drone is equipped with six 1/2.9-inch, 2 MP CMOS sensors. To cover the approximately 147-hectare study site, the flight plan was divided into four polygons, with the altitude ranging from 80 to 110 m. The acquisition parameters for this setup are summarized in Table 1. This configuration produced high-resolution imagery, achieving an average Ground Sample Distance (GSD) of approximately 5 cm per pixel, which allowed for the effective capture of *P. velutinum* infestations under representative environmental conditions. In total, 2565 images were collected, each consisting of five spectral bands: blue, green, red, red-edge, and near-infrared. To address inter-band displacement resulting from shutter lag, the spectral bands were co-registered using the Speeded-Up Robust Features (SURF) algorithm.

A carefully selected non-redundant subset of images, showcasing diverse spatial features and forest landscapes, was used to create a labeled classification dataset instead of a georeferenced cartographic product. Forestry experts manually segmented and labeled the images based on specific phenotypic traits of *P. velutinum*, particularly its dense branching clusters in the upper canopy, oval-shaped leaves, and distinctive yellowish-green coloration. To ensure the accuracy of the labels, the expert annotations were verified against ground-truth points for mistletoe infestations, which were collected during fieldwork and provided by the Commission on Natural Resources and Rural Development (Comisión de Recursos Naturales y Desarrollo Rural, CORENA).

**Figure 6:**
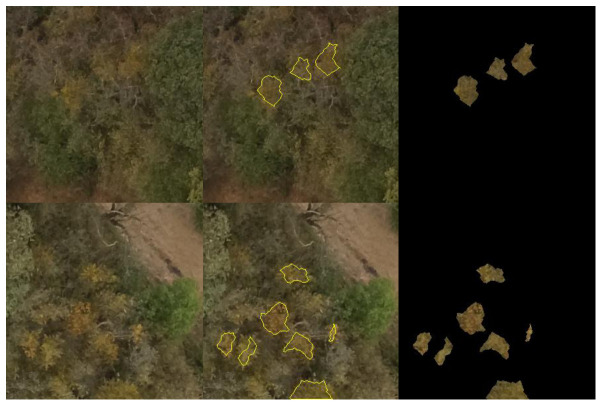
Representative canopy regions infested with *P. velutinum* (rows 1 and 2) showing: original imagery (column 1), expert labeling (yellow contours in column 2), and isolated infested regions for visual clarity (column 3).

Figure 6 illustrates two examples of manual photo-interpretation and expert segmentation for trees infested with *P. velutinum*. Detailed information regarding the raw image collection and the pre-processing workflow is available in [9]. The curated dataset, comprising multispectral tiles, RGB composites, and binary segmentation masks, is publicly accessible at [38].

To prevent data contamination and eliminate local texture redundancy, data partitioning was strictly applied at the source scene level before any cropping operations. The original set of 250 high-resolution images was divided into disjoint subsets: 80% (200 images) for development and 20% (50 images) for independent testing. From the development pool, a subset of 10% (20 images) was reserved for validation, leaving 180 images exclusively for network training.

Subsequently, a total of 5000 sub-image patches were generated within their designated subsets. This restriction ensures that adjacent patches from the same continuous structure are not distributed across different subsets, resulting in exactly 3600 training instances, 400 validation instances, and 1000 test instances. This strict separation guarantees that the testing set serves as a benchmark for entirely unseen visual domains, allowing for an accurate evaluation of the generalization capability of the discovered network topologies.

A total of 3600 training instances were used during the NAS stage to optimize the CNN architectures. Meanwhile, 400 validation instances were utilized to monitor overfitting and assess performance across different epochs. As a result, the independent testing set, comprising 1000 instances, remained completely unseen during both the NAS and training phases. This testing set was only introduced after the optimization process concluded in order to evaluate the final classification performance of the solutions identified by each strategy used in the comparison.

**Figure 7:**
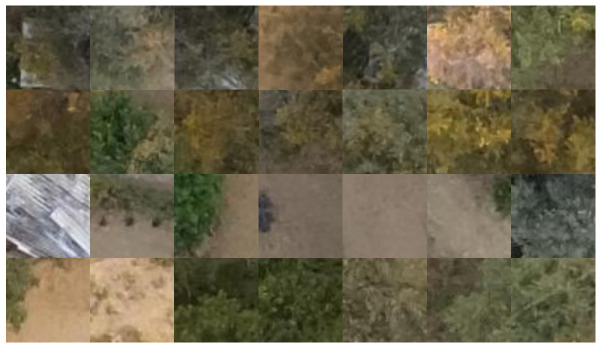
Representative 64×64 pixel patches for network evaluation. Rows 1 and 2 show positive instances of *P. velutinum* with characteristic clustered geometry and dense foliage. Rows 3 and 4 display negative instances with healthy tree crowns and background vegetation, as confirmed by forestry specialists.

Figure 7 presents representative examples of both positive and negative image patches used in this study. In the positive instances (Rows 1–2), the target parasite appears as dense, clustered spherical shapes with fine textural variations, distinguishing it from the linear branch structures of the host. In contrast, the negative samples (Rows 3–4) show healthy foliage, deep canopy shadows, and background elements that define the baseline conditions.

The P4 multispectral sensor captures five key bands: blue, green, red, red-edge, and near-infrared. However, this work only uses the RGB channels. This choice is based on the fact that standard RGB sensors are easy to find and cheaper than specialized multispectral equipment. By optimizing CNN architectures for RGB images, the chances of using and launching these models are improved. This supports local organizations and forest management teams in creating effective monitoring systems with affordable commercial tools.

All computational experiments were implemented using the MATLAB R2025B platform and conducted on a hardware setup detailed in Table 2.

## 4. Results

In the initial stage of the NAS task, the MOEA/D framework was set up using the hyperparameter values outlined in Table 3. These parameters were chosen to optimize the balance between computational budget, convergence, and the diversity of the resulting Pareto-approximate front.

The configuration described above was implemented to perform the simultaneous minimization of two objective functions, $f_{1}$ and $f_{2}$, which are defined as follows:

- $f_{1}=1-$Accuracy;
- $f_{2}=$ Number of trainable parameters.

In the second stage, three representative solutions were selected from the Pareto-approximate front generated by the MOEA/D and passed to the Iterated Local Search (ILS) framework for local refinement. During this local search phase, the optimization continues to focus on the same objective functions, $f_{1}$ and $f_{2}$. However, instead of exploring the broader Pareto trade-off, both objectives are minimized simultaneously. A strict Pareto dominance criterion is used to guide the search toward the local optimum. The configuration of the ILS hyperparameters is detailed in Table 4.

Allocating a proxy budget of 10 epochs for the MOEA/D and ILS optimization helps to avoid the high computational costs associated with design space exploration. While this approach differs from the final evaluation of 500 epochs and may introduce some risks to rank consistency, the 10-epoch proxy is effective for localized representations of 64×64 pixels. Early in the training process, issues such as severe structural bottlenecks, representation collapse, or premature saturation become evident. This allows algorithms to efficiently eliminate subpar topologies.

### 4.1. NAS Results

**Figure 8:**
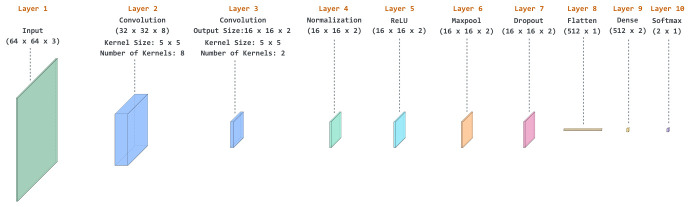
CNN architecture discovered by the hybrid MOEA/D–ILS method, consisting of 10 layers and 2040 trainable parameters.

After completing the NAS stage, the hybrid MOEA/D–ILS framework identified an optimized CNN architecture consisting of 10 layers and 2040 trainable parameters. Figure 8 demonstrates the resulting network topology.

As shown in Figure 8, the CNN architecture processes an RGB input tensor with dimensions of 64×64×3. Two consecutive convolutional layers, each with a stride of 2, progressively reduce the spatial dimensions to 32×32 and then to 16×16 in layers 2 and 3, respectively. Layers 4 and 5 perform normalization and apply the ReLU activation function, helping to stabilize the distribution of activations and introducing non-linearity by eliminating negative values.

The sixth layer employs max pooling to downsample the tensor while retaining the most significant local features. Next, layer 7 introduces a dropout operation to reduce the risk of overfitting. Layer 8 flattens the spatial feature maps into a one-dimensional vector, which is then fed into the fully connected layer (layer 9). This layer consists of two neurons that represent the target classes. Finally, a softmax activation layer calculates the normalized classification probabilities.

### 4.2. Performance Results

To evaluated the structural stability and classification performance of the developed CNN architecture, the model was thoroughly trained across 30 independent trials, with each trial consisting of 500 epochs. Instead of using cross-validation, a holdout validation scheme was implemented, utilizing 400 randomly selected instances. This decision was made due to the high sample density of the database and the significant computational overhead associated with performing iterative cross-validation folds.

Table 5 displays the statistical analysis of the results obtained by the proposed method in comparison to alternative evolutionary search strategies using the same hyperparameter conditions.

**Figure 9:**
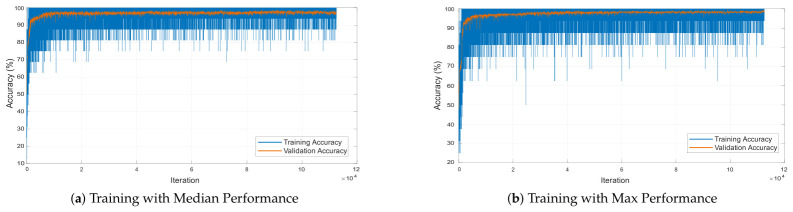
CNN validation accuracy trajectories over 500 training epochs: (**a**) trial yielding median performance; (**b**) trial yielding maximum performance.

Table 5 shows that the proposed framework overextended the classification performance of the baseline algorithms while also maintaining a significantly smaller parameter footprint. Additionally, Figure 9 displays the median and maximum validation accuracy trajectories throughout the training epochs.

As shown in the convergence profiles (Figure 9), training the model for 500 epochs was sufficient to achieve stable asymptotic validation behavior. Given the mini-batch constraints, the network required exactly $1.125\times 10^{5}$ discrete optimization updates to complete the full execution, computed as follows:

$$total\text{\_}iters=\frac{train\text{\_}size}{mini\text{\_}batch\text{\_}size}\times num\text{\_}epochs=\frac{3600}{16}\times 500=11.25\times 10^{4}$$

Following the completion of the exhaustive training phase, we evaluated the generalization capabilities of the optimized CNN model using a separate testing set. To ensure an unbiased assessment, all baseline models were trained from scratch under the same conditions, without the use of transfer learning or external weights. Additionally, an initial resizing layer was integrated into each baseline workflow to preserve their native input layer dimensions without altering the underlying network architectures. High-capacity deep architectures (such as ResNet50, VGG16, or Inception-v3) trained from scratch under data-constrained conditions may struggle with generalization due to the risk of overfitting. In contrast, lightweight topologies are inherently better suited for these operational limits. Therefore, the comparative results provide empirical evidence of their effectiveness within this specific experimental context, rather than making an absolute claim of superiority over deep models in unrestricted environments or pre-trained setups. The comparative performance benchmark is presented in Table 6.

**Figure 10:**
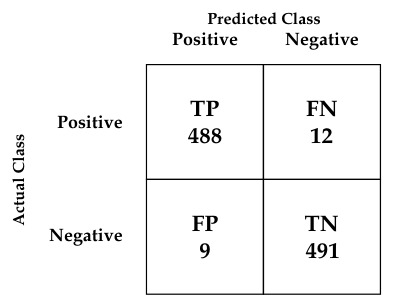
Confusion Matrix (TP = 488, TN = 491, FP = 9, FN = 12) achieved by the proposed method.

The quantitative data presented in Table 6 clearly demonstrate that the optimized CNN architecture, developed through a combined MOEA/D–ILS exploration, outperformed all baseline methods across all evaluated metrics. For a detailed look at the classification boundary behavior, the testing confusion matrix is shown in Figure 10.

The model misclassified only 21 out of 1000 testing instances, including 9 false positives and 12 false negatives, as shown in Figure 10. A visual inspection reveals that the 12 false negatives occur exclusively in regions with high spectral and chromatic similarity, where the target parasite foliage blends into the host canopy background or is partially obscured. Conversely, the 9 false positives are triggered by localized visual ambiguities, such as deep shadows casting sharp features or senescent vegetation that mimics the mistletoe’s cluster geometry. This pattern of localized errors confirms that the discovered network effectively identifies actual *P. velutinum* structures, demonstrating high specificity and avoiding responses to generalized environmental noise.

Additionally, Table 7 displays the average training and classification processing times for each framework investigated.

As shown in Table 7, there is a clear correlation between the complexity of CNN architectures and the computational overhead needed for both training and single-instance inference. The CNN model developed using the proposed methodology features a streamlined architecture, resulting in the lowest computational footprint. Specifically, this optimized network has a total storage size of only 7.97 KB on disk, assuming standard 32-bit floating-point precision. It requires approximately 1.44 MFLOPs per inference cycle, thereby enhancing both training speed and deployment efficiency on hardware-constrained platforms.

**Figure 11:**
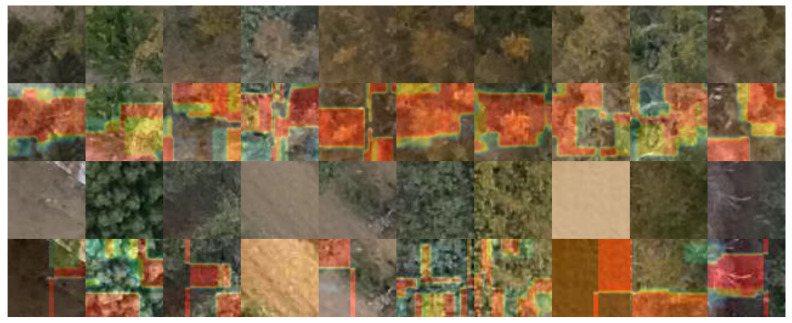
Grad-CAM activations highlighting regions of maximum (red), intermediate (yellow), minimum (blue) and null (uncolored) influence on the classification decision. Row 1 display positive mistletoe instances with their corresponding heatmaps in row 2; row 3 display negative instances with their corresponding heatmaps in row 4.

To verify how this optimized topology uses its structural parameters to isolate target features, a representative subset of sample images, along with their corresponding Gradient-weighted Class Activation Mapping (Grad-CAM) visualizations, is presented in Figure 11.

**Figure 12:**
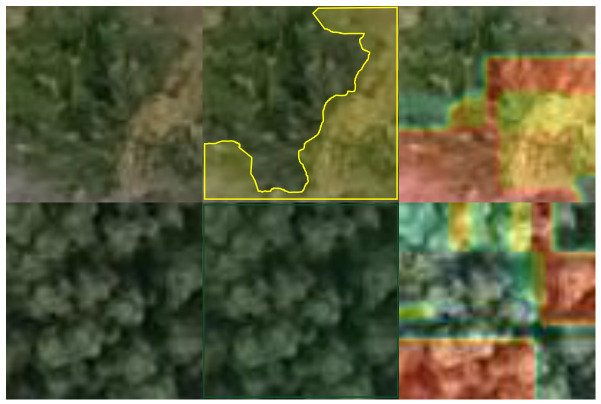
Feature localization comparison against expert knowledge. The first row presents a positive *P. velutinum* instance, showcasing the original patch (first column), the expert-delineated ground truth boundary (yellow label in second column), and the predicted Grad-CAM heatmap (third column). Correspondingly, the second row illustrates a negative instance under identical structural juxtaposition, demonstrating how activations are strictly confined to non-target background features in the absence of the parasite. In the Grad-CAM heatmaps, the red, yellow, and blue colors represent regions of maximum, intermediate, and minimum influence on the classification decision, respectively; uncolored regions exert no influence on the final classification result.

To enhance this analysis, Figure 12 presents representative positive and negative cases alongside their corresponding expert-defined ground-truth boundaries (in yellow) and the predicted activation maps. This side-by-side comparison reveals that the network translates its learned weights into spatial heatmaps that closely match the physical boundaries identified by forestry specialists. This visual agreement highlights the model’s ability to generalize and its high specificity, even in the complex environments of forest canopies.

## 5. Conclusions

This paper presents a hybrid multi-objective evolutionary framework for automatically generating lightweight convolutional neural network (CNN) architectures specifically for classifying *P. velutinum* mistletoe using sub-image patches derived from UAV (Unmanned Aerial Vehicle) imagery. The methodology consists of two sequential stages: a macro-exploration phase that employs MOEA/D (Multi-Objective Evolutionary Algorithm based on Decomposition) to simultaneously minimize classification errors and the number of trainable parameters, followed by a fine-tuning phase utilizing an ILS. To connect these two approaches, the ILS modifies its single-objective nature by applying a strict Pareto dominance criterion rather than conventional scalarization. This ensures that a candidate topology is accepted only if it improves at least one objective function without compromising the other.

The proposed method resulted in an optimized CNN architecture that comprises 10 layers and only 2040 trainable parameters. This architecture significantly outperforms state-of-the-art models in terms of structural simplicity. An independent evaluation showed robust performance across various metrics, achieving an accuracy of 0.979, precision of 0.982, recall of 0.976, F1-score of 0.979, and a Jaccard similarity coefficient of 0.958. Additionally, the computational overhead was greatly reduced, requiring only 67 min for complete training and 1.33 milliseconds for single-instance inference.

These findings clearly demonstrate the significant potential of multi-objective neural architecture search (NAS) for lightweight vegetation monitoring. However, a balanced interpretation indicates that these results are currently limited by the data evaluated. A primary limitation of the discovered model is that its generalization across different geographical locations, seasonal acquisition periods, diverse forest conditions, and various imaging configurations has not been verified. Therefore, extensive validation in a range of ecological systems is necessary before considering large-scale operational deployment. Future research will focus on expanding the architectural representation to handle dynamic layer configurations and on quantifying uncertainty propagation. This will enable the seamless integration of this patch-level inference engine into automated regional forest management workflows.

## Figures and Tables

**Table 1:** Flight polygon parameters for UAV image acquisition over San Bartolo Ameyalco on 13 April 2021.

Polygon	Area (ha)	Altitude (m)	GSD (cm/px)
1	31.40	110	5.8
2	46.34	80	4.2
3	49.14	90	4.8
4	20.37	110	5.8
Total	147.25	—	4.2–5.8

**Table 2:** Specifications of the hardware used for all experiments.

**CPU Specifications**	
Processor:	Intel Core i7-9700K at 3.60 GHz
RAM:	16 GB
**GPU Specifications**	
Model:	NVidia Titan RTX
VRAM:	24 GB DDR5

**Table 3:** Hyperparameter configurations for the MOEA/D framework and CNN training within the initial NAS stage.

MOEA/D	CNN Training Options
Population Size: 50	Max. Epochs: 10
Max. Generations: 100	Learning Rate: $1\times 10^{-3}$
T (number of neighbors): 8	Optimizer: Adam
Crossover Points: 1	Validation Frequency: 30
Mutation Probability: 0.3	MiniBatchSize: 16
	Shuffle: Every epoch
	Data Augmentation: Limited to patch extraction via overlapping windowing; no synthetic transformations (e.g., rotations or pixel alterations) were applied.

**Table 4:** Hyperparameter configurations for the ILS refinement stage and CNN training.

ILS	CNN Training Options
Max. Iters: 1000	Max. Epochs: 10
Perturb Size: [1,3]	Learning Rate: $1\times 10^{-3}$
Acceptation Criterion: Better Solution	Optimizer: Adam
	Validation Frequency: 30
	MiniBatchSize: 16
	Shuffle: Every epoch
	Data Augmentation: Limited to patch extraction via overlapping windowing; no synthetic transformations (e.g., rotations or pixel alterations) were applied.

**Table 5:** Statistical performance and model complexity comparison across 30 independent trials (500 epochs). Training metrics reflect a holdout validation scheme using 400 randomly selected instances. The NTP is expressed in thousands (K).

Method	Min	Max	Median	Avg	StdDev	NTP
MOPSO [39]	0.80	0.85	0.83	0.83	0.02	16.6 K
NSGA-II [16]	0.81	0.84	0.83	0.83	0.01	15.2 K
MOEA/D [40]	0.81	0.83	0.82	0.82	0.01	12.0 K
Hybrid MOPSO	0.89	0.96	0.93	0.94	0.02	3.7 K
Hybrid NSGA-II	0.92	0.97	0.95	0.95	0.02	2.6 K
Proposed Method	0.97	0.99	0.98	0.98	0.01	2.0 K

**Table 6:** Classification performance and complexity comparison against established CNN architectures using 1000 independent testing instances. Metrics include Accuracy (Acc), Precision (Prec), Recall, F1-Score (F1), and Jaccard Similarity Coefficient (JSC). The Number of Trainable Parameters (NTP) is denoted in thousands (K) or millions (M).

Method	Acc	Pres	Recall	F1	JSC	NTP
ResNet50 [34]	0.912	0.922	0.900	0.911	0.836	25 M
Inception-v3 [41]	0.890	0.933	0.840	0.884	0.792	23.8 M
VGG-16 [32]	0.930	0.939	0.920	0.929	0.867	138 M
MobNetV2 [12]	0.860	0.875	0.840	0.857	0.750	2.2 M
EffNetB0 [13]	0.860	0.891	0.820	0.854	0.745	4 M
Franco et al. [26]	0.927	0.926	0.928	0.927	0.864	19 M
MOPSO [39]	0.797	0.810	0.776	0.793	0.656	16.6 K
NSGA-II [16]	0.791	0.795	0.784	0.790	0.658	15.2 K
MOEA/D [40]	0.792	0.786	0.802	0.794	0.658	12 K
Hybrid MOPSO	0.957	0.967	0.946	0.957	0.916	3.7 K
Hybrid NSGA-II	0.971	0.976	0.966	0.971	0.943	2.6 K
Proposed method	0.979	0.982	0.976	0.979	0.958	2 K

**Table 7:** Average time needed for training and classifying a single instance by different CNN models, including the one found using the proposed method, with 500 epochs and a mini-batch size of 16 instances.

CNN Model	Training (min)	Single Classification (ms)
ResNet50 [34]	200	5.30
Inception-v3 [41]	280	10.60
VGG-16 [32]	333	13.30
MobNetV2 [12]	80	1.75
EffNetB0 [13]	102	4.20
Franco et al. [26]	122	5.30
MOPSO [39]	96	1.48
NSGA-II [16]	94	1.47
MOEA/D [40]	92	1.47
Hybrid MOPSO	71	1.36
Hybrid NSGA-II	69	1.34
Proposed method	67	1.33

## Data Availability

The necessary image database and the Matlab code to replicate the achieved performance results can be found at https://github.com/mgil-utleon/mistletoeclassification (accessed on 22 May 2026).

## Abbreviations

The following abbreviations are used in this manuscript:

CNN	Convolutional Neural Network
FLOP	Floating Point Operation
FN	False-Negative
FP	False-Positive
ILS	Iterated Local Search
ITC	Individual Tree Crown
MOEA/D	Multi-Objective Evolutionary Algorithm based on Decomposition
NAS	Neural Architecture Search
NSGA-II	Non-Dominated Sorting Genetic Algorithm Version 2
TN	True-Negative
TP	True-Positive
UAV	Unmanned Aerial Vehicle
